# Leveraging Artificial Occluded Samples for Data Augmentation in Human Activity Recognition

**DOI:** 10.3390/s25041163

**Published:** 2025-02-14

**Authors:** Eirini Mathe, Ioannis Vernikos, Evaggelos Spyrou, Phivos Mylonas

**Affiliations:** 1 Department of Informatics and Computer Engineering, University of West Attica, 12243 Athens, Greece; emathe@uniwa.gr (E.M.); mylonasf@uniwa.gr (P.M.); 2 Department of Informatics and Telecommunications, University of Thessaly, 35100 Lamia, Greece; ivernikos@uth.gr

**Keywords:** human activity recognition, data augmentation, occlusion

## Abstract

A significant challenge in human activity recognition lies in the limited size and diversity of training datasets, which can lead to overfitting and the poor generalization of deep learning models. Common solutions include data augmentation and transfer learning. This paper introduces a novel data augmentation method that simulates occlusion by artificially removing body parts from skeleton representations in training datasets. This contrasts with previous approaches that focused on augmenting data with rotated skeletons. The proposed method increases dataset size and diversity, enabling models to handle a broader range of scenarios. Occlusion, a common challenge in real-world HAR, occurs when body parts or external objects block visibility, disrupting activity recognition. By leveraging artificially occluded samples, the proposed methodology enhances model robustness, leading to improved recognition performance, even on non-occluded activities.

## 1. Introduction

Human activity recognition (HAR) is a contemporary research field that intersects key areas, including computer vision, machine learning, and signal processing. The primary objective is the automated identification of human activities based on a series of observations in the temporal and/or spatial domain. This typically entails the detection and recognition of gestures, postures, or movements of the human body, followed by the interpretation of the observed activities. Sensors play a crucial role in this process and can be wearable or installed in the user’s environment [1]. These sensors capture and collect data, including visual, auditory, or motion information, which are then processed to the enable automated recognition of human activities.

Recent research in human activity recognition (HAR) has predominantly focused on leveraging deep learning techniques [2] to infer conclusions about a subject’s activity. Typical deep HAR approaches follow a generic methodology. The process begins with a subject engaging in an activity (e.g., climbing stairs), and sensors capture measurements related to the motion. The captured measurements undergo processing so as to be used as input to deep trained networks, which analyze data to classify the subject’s activity. HAR approaches may utilize different types of sensors providing various types of data, and the choice depends on factors such as accuracy, cost, power consumption, and ease of integration. Wearable sensors include smartwatches, body-worn sensors, and smartphones, while environmental sensors encompass video/thermal cameras, microphones, infrared, pressure, magnetic, and RFID sensors [3]. However, wearable sensors are not preferred by users due to usability issues [4,5], and overloading environments with multiple sensors can be expensive and intrusive. Consequently, low-cost solutions often rely solely on cameras that detect activities using the subject’s captured motion.

As is well known, one of the main challenges in recognition tasks approached using deep learning architectures is the limited size of the available training datasets [6]. Specifically, using small datasets to train deep learning models can lead to overfitting, where models learn noise and fail to generalize to new data. The limited diversity in these datasets may not capture real-world variability, resulting in poorer model performance and increased output variance. Furthermore, small datasets often provide insufficient information for both deep learning models and robust model evaluation, leading to oversimplified models that do not accurately represent complex behaviors. To mitigate these issues, several approaches can be applied, such as data augmentation, transfer learning, or even the use of simpler models.

In this paper, we propose a data augmentation technique that is based on the creation of artificially occluded samples of activities. Specifically, contrary to previous work [7], where we augmented the training dataset by incorporating artificially rotated skeletons within the training process, in this one, we artificially remove body parts from the skeletons so as to simulate the effect of occlusion. This approach significantly enhances the training dataset in terms of both size and diversity, enabling the network to learn from a broader range of examples.

In the context of human activity recognition (HAR), occlusion refers to the partial or complete blocking of a person, which can prevent the activity from being fully visible and accurately recognized by a trained recognition model [8]. Possible causes of this effect include external objects, such as furniture or other people present in the scene (commonly referred to as “external occlusion”), or the subject’s own body parts, for example, when one arm obscures the other during an action (referred to as “self-occlusion”). Both types of occlusion disrupt the continuity of motion and obscure the vital visual cues necessary for the accurate classification of actions. This challenge is particularly prominent in real-world scenarios where occlusion frequently occurs [8,9]. The impact of occlusion varies based on its extent, duration, and the importance of the occluded body parts to the action being performed [9]. For example, in an activity such as “kicking something”, the movement of the legs is crucial, and their occlusion can lead to significant errors in recognition. In contrast, the same activity may still be recognizable even with the occlusion of both arms.

As the field of human activity recognition continues to evolve, addressing occlusion effectively remains a key area of research. The development of more sophisticated models that can handle various types of occlusions not only improves the accuracy of action recognition systems but also expands their applicability to real-world environments where occlusions are common. In this work, however, we attempt to exploit the effect of occlusion as a means of training more robust recognition models. Unlike typical research that focuses on overcoming occlusions in test scenarios, our novel methodology uses artificially occluded samples during training to augment the dataset, with the goal of improving performance on *non-occluded* samples.

The remainder of this paper is organized as follows: In Section 2, we discuss related work regarding occlusion and data augmentation in the context of HAR. Section 3 presents the proposed methodology for data augmentation using artificially created occluded samples. The evaluation of the proposed approach and the corresponding results are discussed in Section 4. Finally, conclusions are drawn in Section 5.

## 2. Related Work

### 2.1. Occlusion in HAR

Recent research on occlusion in human activity recognition (HAR) has increasingly focused on identifying activities even when one or more body parts are not fully visible. For example, Giannakos et al. [8] investigated the effect of occlusion on HAR by artificially creating occluded skeletons. They achieved this by selectively removing structured body parts, such as arms and legs, before classification to assess how these occlusions affect recognition performance. Building on this body of work, in the following, additional studies that address the challenges of occlusion in the context of HAR are presented. These studies primarily explore various methods of simulating or handling the partial visibility of body or skeleton parts to improve the robustness of activity recognition systems.

Many existing research efforts in human activity recognition (HAR) rely on extracted skeleton data. To the best of our knowledge, no publicly available datasets contain naturally occluded samples. Consequently, most research studies generate artificially occluded data to evaluate and improve their methods. One common approach for creating such data involves the removal of structured body parts from skeletons as proposed by Giannakos et al. [8].

Angelini et al. [10] investigated both persistent and short-term occlusion scenarios, simulating occlusions by removing structured sets of skeleton joints that correspond to body parts. To address persistent occlusions, they utilized action prototypes to fill missing information, while for short-term occlusions, they employed interpolation techniques. Similarly, Ghafoor et al. [11] experimented with random and structured occlusions and proposed a temporal dilated convolutional neural network (CNN) that leverages temporal information to estimate missing joints. Vernikos et al. [9] introduced a CNN-based method trained on two-dimensional (2D) representations of three-dimensional (3D) skeletal motion, which included artificially occluded samples in the training process. Their findings demonstrated that incorporating these occluded data samples significantly improved the model’s performance in recognizing activities under conditions where structured body parts were not visible. Lastly, Yang et al. [12] conceptualized the body skeleton as a graph and simulated occlusion by removing sub-graphs. They developed a novel augmentation methodology based on Graph Convolutional Networks (GCNs) to simulate occlusions during training.

In addition to generating artificially occluded skeleton data, other research approaches have focused on using still images and videos containing occluded body parts before extracting skeleton joint information. Bian et al. [13] handled occlusions in a multi-camera setup where specific skeletal parts were not visible in certain camera angles. They introduced a novel learning method to develop view-invariant representations, robust to such occlusions. Li et al. [14] extracted skeletons from images with occluded body parts and introduced a methodology that tackles the occlusion challenge as a missing value imputation process within a feature matrix. Similarly, Gu et al. [15] simulated occlusions by generating artificial masks and applied temporal gated convolutions to reconstruct missing body information. Cheltha et al. [16] utilized images depicting partially occluded human subjects to extract incomplete skeletons and addressed these occlusions by incorporating multiple hypothesis tracking and recurrent neural networks. Meanwhile, Iosifidis et al. [17] implemented a multi-camera setup surrounding the subject, based on the assumption that occlusions would not simultaneously impact all camera views. Lastly, Li et al. [18] utilized depth images and an action graph to model the action dynamics, combined with bag-of-3D points to represent postures. They simulated occlusions by disregarding specific body areas.

### 2.2. Data Augmentation for HAR

Moreover, in recent years, numerous research studies have focused on data augmentation methods specifically designed for human activity recognition (HAR) using extracted skeleton data. In this section, we review related work that employs data augmentation techniques on skeleton information. We begin with approaches applied to the spatial dimension of skeletal data. Chen et al. [19] proposed a simple augmentation strategy that includes scaling, translation, rotation, and the addition of random noise. Similarly, Park et al. [20] and Wang et al. [21] applied rotations to generate artificial viewpoints of skeleton data. Rao et al. [22] extended these augmentation techniques by incorporating rotations, shears, reversals (“flips”), Gaussian noise, Gaussian blur, and partial masking applied either to sets of joints or individual joint coordinates. Additionally, Li et al. [23] utilized 3D rotations and Gaussian noise as part of their data augmentation approach for skeleton-based HAR.

In addition to spatial augmentation approaches, several studies have explored data augmentation techniques focusing on the temporal dimension of skeleton sequences. Xin et al. [24] proposed various temporal data augmentation techniques such as time reversing, interpolation, frame shifting, and temporal warping to introduce variations in action timing. Li et al. [23] employed video cropping to alter the temporal context of actions, effectively trimming sequences to create diverse activity durations. Chen et al. [19] utilized interpolation to simulate activities performed at different speeds, thereby generating sequences of varying lengths as though performed by different actors. Huynh-The et al. [25] introduced random frame elimination and addition to create artificial activity samples of different durations, thus further diversifying the temporal patterns presented to the model.

In recent years, numerous studies have utilized generative architectures, such as generative adversarial networks (GANs), to augment training datasets with artificial skeleton samples and sequences. For instance, Tu et al. [26] and Meng et al. [27] developed LSTM-based generative models capable of creating skeleton sequences that closely resemble real data. Shen et al. [28] introduced the Imaginative GAN, a model designed to approximate the underlying distribution of input skeletal data and generate new skeleton sequences from this learned distribution. Additionally, Wang et al. [29] proposed a method leveraging contrastive learning, utilizing both skeletal coordinates and velocities to produce augmented skeleton sequences that enhance the diversity of the training data.

The proposed approach described in this paper is partially inspired by the works of Vernikos et al. [30] and Angelini et al. [10], both of which included occluded samples in the training process to improve classification performance on occluded data. However, unlike these approaches, our goal is to use data augmentation techniques with artificially occluded samples to enhance classification performance on *non-occluded* samples.

## 3. Methodology

Building upon previous works [30,31,32], our proposed data augmentation training strategy utilizes 3D trajectories of human skeletons as input, which are ultimately represented as activity images created using the discrete sine transform (DST) (see Section 3.1). However, unlike earlier approaches, which were based on the inclusion of modified training samples while keeping the set of joints intact, the novelty of the herein presented work is the inclusion of artificially occluded samples in the training process—that is, samples where structured sets of joints corresponding to specific body parts have been removed, i.e., the set of joints has been reduced.

### 3.1. Extraction and Representation of Skeletal Data

**Figure 1:**
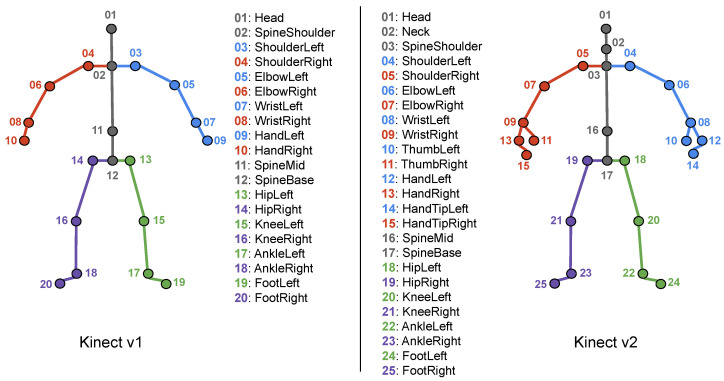
A human body pose with the 20 and 25 skeletal joints that are extracted using the Microsoft Kinect v1 (**left**) and v2 (**right**) cameras. Joints have been divided into subsets, each corresponding to one of the five main body parts, i.e., torso (grey), left arm (blue), right arm (red), left leg (green) and right leg (purple). For illustrative purposes and also to facilitate comparisons between the two different versions, body parts have been colored using the same colors. Numbering follows the Kinect SDK in both cases, therefore there exist several differences between the two versions.

The Microsoft Kinect sensor [33] has played a significant role in revolutionizing human–computer interaction and 3D sensing technology. It was first released in 2010 by Microsoft, initially for use with the XBOX 360 game console, and within its lifetime, two versions, namely Kinect v1 and Kinect v2, have been presented. In the context of this work, the Microsoft Kinect is used to capture the raw skeletal joint motion data. Specifically, the Kinect sensor is capable of extracting the 3D positions (i.e., *x*, *y*, and *z* coordinates) of a human’s skeletal joints in real time using its SDK. Furthermore, a structured graph of joints is continuously streamed, where graph nodes correspond to the most representative body parts (e.g., skeletal joints of arms, legs, head, etc.), and graph edges follow the anatomical structure of the human body. A parent–child relationship is implied from top to bottom; for example, *Head* is the parent of *SpineShoulder*, which is the parent of *ShoulderLeft* and *ShoulderRight*, and so on. In Figure 1, we illustrate the 25 human skeleton joints that are extracted using the Kinect SDK and the v2 sensor; as observed, the latter provides more comprehensive information about the skeletal joints.

In the context of this work, an activity is defined as the transfer of a subset of joints from point *A* to point *B* along a trajectory. To provide a description of such an activity and inspired by the work of Jiang and Yin [2], who utilized raw sensor measurements from inertial sensors, we first create “signal” images by concatenating the signals produced by skeletal motion. Specifically, the motion of each skeletal joint in 3D space over time is treated as three independent 1D signals, each corresponding to a coordinate axis. The first step in creating “signal” images is to concatenate the signals produced by skeletal motion. We consider that the motion of each skeletal joint in 3D space over time is treated as three independent 1D signals, each corresponding to a coordinate axis. Therefore, for a given joint *j*, let $S_{j,x}\left(n\right)$, $S_{j,y}\left(n\right)$, and $S_{j,z}\left(n\right)$ denote the three 1D signals that correspond to its motion along the *x*, *y*, and *z* coordinates, and at the n−th frame, respectively. In the signal image, $S_{j,x}\left(n\right)$ corresponds to row 3×j−2. Accordingly, $S_{j,y}\left(n\right)$ and $S_{j,z}\left(n\right)$ correspond to rows 3×j−1 and 3×j, respectively. In this way, the signal image S for a given activity and *N* joints is created by concatenating the 3×N signals, resulting in a dimension of $3N\times T_{s}$, where $T_{s}$ is the duration of the activity.

It is important to emphasize that our focus lies solely on classifying activities into a predefined set of categories. This means that we do not address the task of identifying the starting and ending frames of a given activity in a video. Instead, we treat this segmentation challenge as already resolved. Consequently, our approach operates on pre-segmented video clips, aiming to identify the activity present in each segment, with the assumption that each segment contains at most one activity. Typically, when using publicly available datasets, such a segmentation is provided.

**Figure 2:**
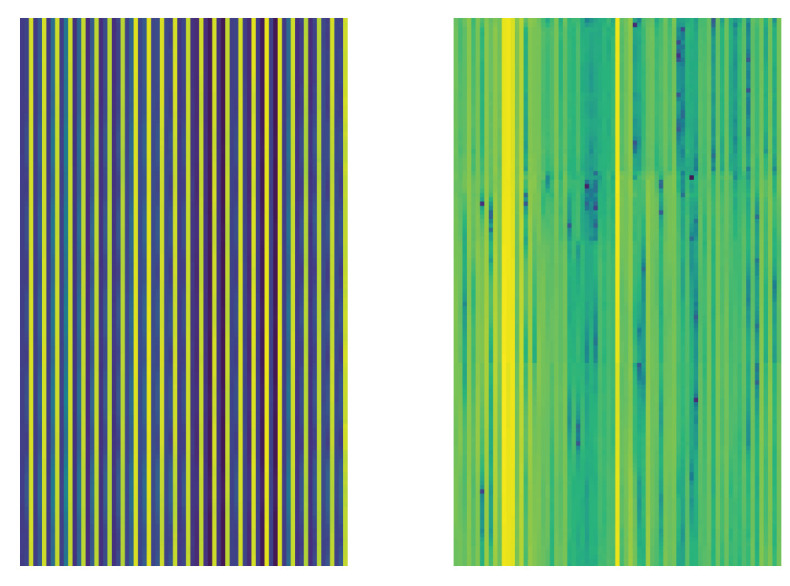
(**left**) a sample signal image; (**right**) the corresponding activity image from activity “hugging other person”. Figure best viewed in color.

However, human activity recognition typically targets real-world scenarios. In such a case, the duration of an activity can vary significantly depending on the individual performing it, and different activities often naturally require different time spans. This introduces variability in segment length, denoted as $T_{s}$. Since the herein proposed method uses a convolutional neural network for classification, its input should have a fixed size. Therefore, to handle the aforementioned inconsistencies and allow the concatenation of signals, we incorporate a linear interpolation step. This ensures that all activity durations are standardized to a fixed length, $T_{a}$. To determine the appropriate value for $T_{a}$, we begin by selecting a duration close to the average length of all activities, refining this value through experimentation and fine-tuning. In our work, $T_{a}$ is set to 159 frames. Additionally, with N=25, the resulting size of S becomes 75×159. An example signal image is illustrated in Figure 2.

For each signal image S, with dimensions W×H, where S(n,m) represents the pixel at coordinates (m,n), an “activity” image A is generated by applying the two-dimensional Discrete Cosine Transform (DCT) [34,35] to S, as defined by:

(1) $$A\left(u,v\right)=a_{u}a_{v}\sum_{x=0}^{W-1}\sum_{y=0}^{H-1}S\left(x,y\right)\left(\cos\frac{\pi (2m+1)u}{2W}\cos\frac{\pi (2n+1)v}{2H}\right)\;,$$

where x∈[0,W−1],y∈[0,H−1],u∈[0,W−1],v∈[0,H−1] and also:

(2) $$a_{u}=\left\{\begin{array}{c} \frac{1}{\sqrt{W}},u=0\\ \sqrt{\frac{2}{W}},1\leq u\leq W-1 \end{array}\right.$$

and

(3) $$a_{v}=\left\{\begin{array}{c} \frac{1}{\sqrt{H}},v=0\\ \sqrt{\frac{2}{H}},1\leq v\leq H-1 \end{array}\right.\;.$$

Thus, A=D(S), where D(·) represents the DCT. It is important to note that only the magnitude of the DCT is retained, while the phase information is discarded. The resulting image is further processed by normalization using the orthonormal basis, yielding a 2-D image with dimensions identical to those of the signal image S. In Figure 2, an example of a signal image from the class “hugging another person” and its corresponding activity image are shown.

### 3.2. Activity Classification

**Figure 3:**
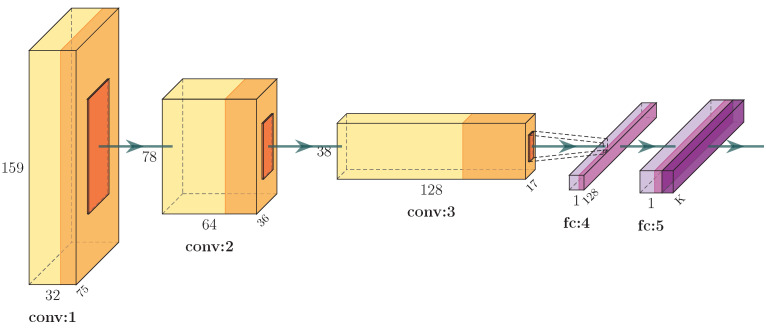
The proposed CNN architecture for recognition of human activities.

The architecture of the proposed CNN is presented in detail in Figure 3. Specifically, the first convolutional layer filters the 159 × 75 input activity image with 32 kernels of size 3 × 3. The first pooling layer uses “max-pooling” to perform 2 × 2 sub-sampling. The second convolutional layer filters the 78 × 36 resulting image with 64 kernels of size 3 × 3. A second pooling layer uses “max-pooling” to perform 2 × 2 sub-sampling. A third convolutional layer filters the 38 × 17 resulting image with 128 kernels of size 3 × 3. A third pooling layer uses “max-pooling” to perform 2 × 2 sub-sampling. Then, a flatten layer transforms the output image of the last pooling to a vector, which is then used as input to a dense layer using dropout. Finally, a second dense layer produces the output of the network. To avoid overfitting, the most popular approach which is also adopted in this work is the use of the dropout regularization technique [36]: at each training stage several nodes are “dropped out” of the network. This way overfitting is reduced or even prevented, since complex co-adaptations on training data are prevented. For training the CNN, the ReLU activation function has been used. Moreover, the batch size is set to 100 and the Adam optimizer is used. Also, the dropout is set to 0.55, the learning rate is set to 0.001 and the network is trained for 43 epochs, using the loss of the validation set calculated via cross-entropy as an early stopping method, in order to avert overfitting.

The aforementioned architecture is selected through validation set tuning based on two factors: (a) the need to build sufficiently rich representations to allow for effective classification; and (b) the restriction of the number of parameters so as to allow flexibility, e.g., for easy deployment of the model in low-cost platforms or mobile devices to perform inference on the edge, in real-life applications of the herein proposed approaches.

### 3.3. Occlusion of Skeletal Data

In real-life scenarios, occlusion is a significant factor that hinders the optimal performance of human activity recognition (HAR) approaches. As described in Section 2.1, several research efforts incorporate occluded samples into the training process to enhance recognition robustness. To elucidate the concept of occlusion, consider, for example, an assisted living scenario where one or more cameras are installed in a subject’s environment to capture their appearance and motion for behavior recognition. In such a setting, the living space may contain furniture or other objects behind which actions can occur or other people may be present, leading to partial or even full occlusion, resulting in a loss of visual information, which can be crucial for recognition even in simple actions. For example, in the activity of “handshaking”, occlusion of the arms would prevent a trained model from recognizing the action.

As previously mentioned, the main goal of this paper is to assess whether the inclusion of occluded samples in the training process—specifically, augmenting the training data with artificially created occluded samples—can improve classification performance. However, publicly available datasets, such as those used in this work’s evaluation (see Section 4), have been created under ideal conditions. That is, the subjects are captured in well-illuminated, empty spaces to ensure full-body visibility, resulting in the absence of occluded samples.

To simulate the effect of occlusion for the experimental evaluation of our work, we follow the paradigms established by previous studies [8,15,30] which indicate that the torso is a relatively rigid part of the body compared to the limbs, and thus most activities are an effect of the motion of arms and/or legs. Moreover, many activities may share a similar torso position but differ in limb movements. Therefore, we remove distinct body parts from the skeleton data—specifically, structured sets of skeleton joints. In the case of the dataset captured using Kinect v2, skeletons comprise 25 joints from which we removed those corresponding to the arms and legs. Specifically, each arm consists of the shoulder, elbow, wrist, hand, thumb, and handtip joints, while each leg includes the hip, knee, ankle, and foot joints. The remaining joints—the head, spine shoulder, spine mid, and spine base—form the torso, which we consider non-occluded in all cases. This decision is supported by our preliminary experiments and aligns with the conclusions of Giannakos et al. [8] and Vernikos et al. [30]. However, in the case of datasets captured using Kinect v1, comprising 20 joints, there are slight differences in the composition of the arms and torso. In such datasets, each arm includes the shoulder, elbow, wrist, and hand joints, while the torso consists of the head, neck, spine shoulder, spine mid, and spine base joints. Despite these minor differences, we form five body parts in both cases as illustrated in Figure 1.

To further simulate occlusion—consistent with the assumptions in [8,30]—we consider occlusion affecting the entire duration of the activity. We intuitively select the following cases of occlusion: (a) one arm; (b) one leg; (c) both arms; (d) both legs; and (e) one arm and one leg on the same side. This results in a total of eight cases. We assume that when two body parts are occluded simultaneously, they belong to the same side of the body. We consider two augmentation scenarios: augmenting the training dataset using samples where (a) one or both arms or an arm and a leg are occluded (resulting in a dataset 6× larger than the original), and (b) all eight aforementioned occlusion cases are included (resulting in a dataset 9× larger than the original).

**Figure 4:**
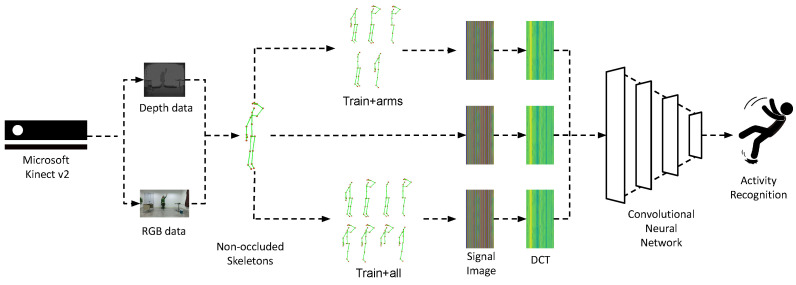
A visual overview of the proposed data augmentation methodology incorporating artificially occluded samples.

**Figure 5:**
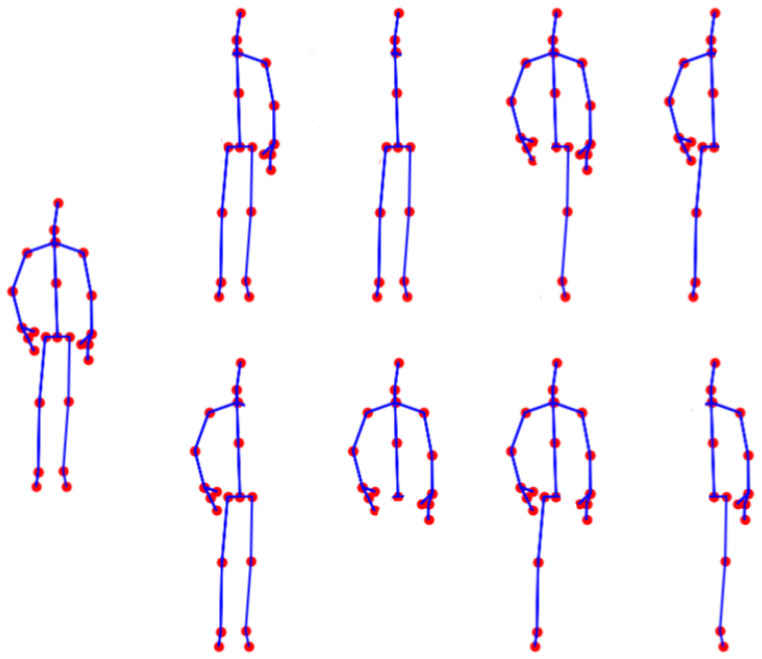
An illustration of a full skeleton and the eight cases of occlusion. The first row displays occlusions of the following: right arm, both arms, right leg, and left arm with left leg. The second row shows occlusions of the following: left arm, both legs, left leg, and right arm with right leg.

**Figure 6:**
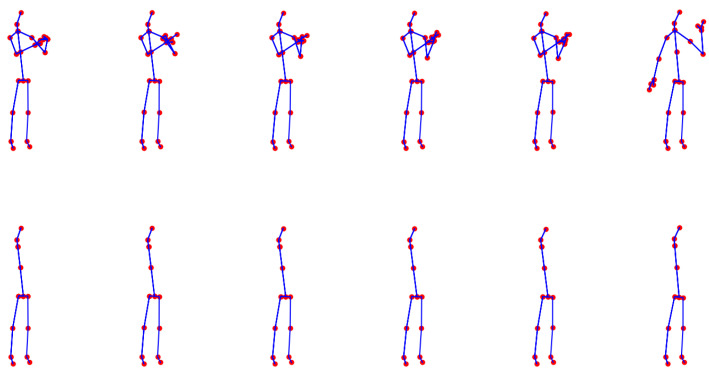
Example skeleton sequences of the activity “make phone call/answer phone” from the PKU-MMD dataset, captured using Microsoft Kinect v2. The first row displays the original skeletons, including all 25 joints (without any occlusion). The second row shows skeletons where joints corresponding to both arms have been removed, illustrating a case of partial occlusion.

A visual overview of the proposed augmentation approach is presented in Figure 4. In Figure 5, we illustrate an example of a full skeleton along with all eight cases of artificial occlusion. Additionally, Figure 6 depicts an example of an activity with the full skeleton and with both arms occluded. This example demonstrates that the occlusion of these two body parts leads to a significant loss of visual information, rendering the performed activity unrecognizable.

## 4. Experimental Protocol and Results

We are not aware of any publicly available datasets that contain real 3D occluded actions. To address this and in order to evaluate the proposed approach, we manually exclude the structured subsets of skeletal joints forming body parts (e.g., arms and legs) from three publicly available datasets, that provide 3D skeletal information. To experimentally evaluate the proposed approach, we utilized the following datasets:

- The PKU-MMD dataset [37] is a publicly available, open-source benchmark for 3D human motion-based activity recognition. From this dataset, we select 11 actions (4538 activity samples) that are closely related to the activities of daily living (ADLs) [38,39], namely, eating, falling, handshaking, hugging, making a phone call, playing with a phone or tablet, reading, sitting down, standing up, typing on a keyboard, and wearing a jacket. In this case, we consider single-view, cross-view, and cross-subject evaluation scenarios [39].
- The SYSU 3D Human–Object Interaction (HOI) dataset [40] focuses on 3D human motion-based interactions between people and objects. It contains 480 activity samples from 12 different activities, specifically: drinking, pouring, calling phone, playing phone, wearing backpacks, packing backpacks, sitting chair, moving chair, taking out wallet, taking from wallet, mopping, and sweeping. The dataset involves 40 subjects interacting with one of the following objects per case: phone, chair, bag, wallet, mop, and besom. Each activity has 40 samples.
- The UTKinect-Action3D dataset [41] includes 10 different activities performed by 10 different subjects, namely walking, sitting down, standing up, picking up, carrying, throwing, pushing, pulling, waving hands, and clapping hands. Each activity is performed twice by each subject, resulting in a total of 200 activity instances.

Note that from the aforementioned datasets, we only use 3D skeleton motion data and disregard other modalities, while in all cases, datasets are split following the protocol imposed by their authors. Also, PKU-MMD is recorded using Microsoft Kinect v2 and under three camera viewpoints, while SYSU-3D-HOI and UTKinect-Action3D are recorded using Microsoft Kinect v1 and under a single camera viewpoint.

For the experimental evaluation of the proposed methodology, we consider the following training strategies:

a. A baseline approach, denoted as “Full”, where the convolutional neural network (CNN) is trained using only non-occluded samples, following the exact methodology presented in [32].
b. Augmentation of the dataset from case [a.], denoted as “Full+Arms”, by including simulated occlusion examples involving the removal of structured sets of skeletal joints corresponding to the arms. The occlusion cases considered are (a) left arm; (b) right arm; (c) both arms; (d) left arm and left leg; and (e) right arm and right leg.
c. Further augmentation of the dataset from case [b.], denoted as “Full+All”, by adding simulated occlusion examples involving the removal of structured sets of skeletal joints corresponding to: (a) left leg; (b) right leg; and (c) both legs.

In all the aforementioned experiments, we evaluate the performance of classifying non-occluded samples using a convolutional neural network (CNN) trained with activity images. The experimental results for all datasets are presented in Table 1, Table 2 and Table 3. In all cases, we report the mean accuracy.

For the PKU-MMD dataset, in the single-view experiments, we observed the following: for the M and R cameras, the augmentation case “+Arms” exhibited the best accuracy in both cases, with values of 0.95 and 0.94, respectively. However, for the L camera, “Full+All” achieved marginally better accuracy than “Full+Arms,” with an accuracy of 0.94. In all cases, the augmentation strategies demonstrated significantly increased accuracy compared to the baseline. Moreover, when comparing the average accuracy across all single-view cases, we can argue that “Full+Arms” demonstrated better overall performance. In the cross-view experiments, in most cases, “Full+All” showed better performance, although in several cases the differences were marginal. Nevertheless, the improvement provided by the augmentation step over the baseline approach was clear in all cases. Comparing the average accuracy across all cross-view cases, we can argue that “Full+Arms” again demonstrated better overall performance. Finally, in the cross-subject experiments, interestingly, “Full+Arms” exhibited the best performance, with an accuracy of 0.88, while “Full+All” was significantly lower than the baseline case.

With the SYSU dataset, in the single-view experiments, we noticed a significant increase in performance. Specifically, in the single-view experiments (Setting 1), without augmentation, the accuracy was 0.58, which increased to 0.69 with the “Full+All” augmentation—exhibiting an improvement of 19%. Similarly, in the cross-subject experiments (Setting 2), without augmentation, the accuracy was 0.56, reaching 0.60 with “Full+Arms”, indicating an increase of 7%. Finally, with the UTKinect-Action3D dataset, without augmentation, the accuracy was 0.85, which rose to 0.90 with “Full+Arms”, showing an improvement of 5.9%. Interestingly, for this dataset, “Full+All” showed equal accuracy to the baseline case.

## 5. Conclusions

In this paper, a data augmentation approach which is based on the artificial occlusion of body parts, and targets the problem of human activity recognition from video data was presented. Specifically, we used 3D skeletons from which we manually removed one or two body parts. We performed experiments where the training data set was firstly augmented using samples where at least one arm was occluded, and we further augmented it using samples where at least one leg was occluded. Experiments have been performed using three datasets of human motion activities, which were recorded with single and multi-camera setups. In the latter case, we conducted a three-fold evaluation, i.e., a single view case where the same viewpoint was used for training/testing, a cross-view case where different viewpoints were used for training/testing and cross-subject cases, where different subjects were used for training/testing. Actions were represented using activity images created by applying the Discrete Sine Transform on raw motion data, and a CNN was trained for each dataset and for each augmentation case. The experimental evaluation indicated that the proposed approach may be successfully used for HAR in most of the aforementioned cases, as it is able to provide a significant performance boost over the baseline approaches.

We believe that the proposed approach could benefit several HAR tasks which take place in dynamic environments. The proposed algorithm could be deployed, for example, within an assisted living environment (e.g., a smart home), for monitoring the activities of daily living (ADLs) [38], or medical-related events that can be recognized by motion and may require some kind of attention or intervention. Another application could be the recognition of human motion within augmented reality environments and applications. In such a case, human motion may offer useful cues for assessing user engagement or satisfaction. The latter could be a possible application in the area of retail and marketing, upon the recognition of behaviors and interactions with products, or in the area of sports and fitness, where it could act as a means of assessing performance. Surveillance and security applications may also benefit, e.g., for the detection of suspicious activities or individuals. Moreover, it could benefit the broader area of human–computer interaction by recognizing gestures and actions to control interfaces or interact with virtual objects or even provide assistive interaction approaches for people with disabilities, or incorporation of player movements into gameplay mechanics, in the area of gaming. Finally, another possible application could the analysis of classroom activities and student engagement within an education environment.

## Figures and Tables

**Table 1:** Results on the PKU-MMD dataset. “Full” denotes the case where the training dataset comprises only non-occluded samples. “Full+Arms” denotes the case where “Full” is augmented with samples in which one or both arms are occluded. “Full+All” denotes the case where “Full+Arms” is further augmented with samples in which arms are always present but one or both legs of the same side are occluded. Numbers denote accuracy, with bold numbers indicating the best performance among the three training strategies. M, L, and R denote activity samples captured by the middle, left, and right cameras, respectively.

Experiment	Train	Test	Full	Full+Arms	Full+All
Single-view	M	M	0.86	**0.95**	0.85
	L	L	0.84	0.87	**0.88**
	R	R	0.87	**0.94**	0.90
Cross-view	M	L	0.64	**0.84**	0.83
	M	R	0.63	0.81	**0.86**
	L	M	0.72	0.81	**0.83**
	L	R	0.43	0.55	**0.56**
	R	M	0.63	0.85	**0.89**
	R	L	0.39	0.40	**0.45**
	M,L	R	0.62	**0.84**	0.82
	M,R	L	0.60	**0.78**	0.77
	L,R	M	0.82	0.94	**0.95**
Cross-subject	M,L,R	M,L,R	0.81	**0.88**	0.75

**Table 2:** Results on the SYSU dataset. “Full” denotes the case where the training dataset comprises only non-occluded samples. “Full+Arms” denotes the case where “Full” is augmented with samples in which one or both arms are occluded. “Full+All” denotes the case where “Full+Arms” is further augmented with samples in which arms are always present, but one or both legs of the same side are occluded. Numbers denote accuracy, with bold numbers indicating the best performance among the three training strategies.

Experiment	Full	Full+Arms	Full+All
Single-view	0.58	0.62	**0.69**
Cross-subject	0.56	0.55	**0.60**

**Table 3:** Results on the UTKinect-3D dataset. “Full” denotes the case where the training dataset comprises only non-occluded samples. “Full+Arms” denotes the case where “Full” is augmented with samples in which one or both arms are occluded. “Full+All” denotes the case where “Full+Arms” is further augmented with samples in which arms are always present, but one or both legs of the same side are occluded. Numbers denote accuracy, with bold numbers indicating the best performance among the three training strategies.

Experiment	Full	Full+Arms	Full+All
Single-view	0.85	**0.90**	0.85

## Data Availability

The PKU-MMD dataset is available at https://www.icst.pku.edu.cn/struct/Projects/PKUMMD.html (accessed on 13 February 2025). The NTU-RGB+D dataset is available at https://rose1.ntu.edu.sg/dataset/actionRecognition/ (accessed on 13 February 2025). The UT-Kinect-3D dataset is available at https://cvrc.ece.utexas.edu/KinectDatasets/HOJ3D.html (accessed on 13 February 2025). The SYSU-3D-HOI dataset is available at https://www.isee-ai.cn/~hujianfang/ProjectJOULE.html (accessed on 13 February 2025).
